# Femtosecond laser-assisted cataract surgery after corneal refractive surgery

**DOI:** 10.1038/s41598-022-08297-8

**Published:** 2022-03-11

**Authors:** Hyunmin Ahn, Ikhyun Jun, Kyoung Yul Seo, Eung Kweon Kim, Tae-im Kim

**Affiliations:** 1 Department of Ophthalmology, Yonsei University College of Medicine, Seoul, Korea; 2 Department of Ophthalmology, Corneal Dystrophy Research Institute, Yonsei University College of Medicine, 50-1 Yonsei-ro, Seodaemungu, Seoul, 03722 Korea; 3 Saevit Eye Hospital, Goyang, Gyunggi-do Korea

**Keywords:** Medical research, Outcomes research

## Abstract

Cataract is the leading cause of blindness worldwide, and advanced cataract techniques such as femtosecond laser-assisted cataract surgery (FLACS) have been commercially available. Corneal refractive surgery (CRS) is one of the most popular surgeries for the correction of refractive errors. CRS changes the cornea not only anatomically but also pathophysiologically. However, there has been no clinical research analyzing the refractive and safety outcomes of FLACS after CRS. The aim of this retrospective chart review and comparative study is to evaluate the effect and safety of FLACS after CRS comparing with conventional PCS. Participants with a previous CRS history who underwent FLACS or conventional PCS were included in this study. The visual outcomes and the refractive outcomes including refractive, corneal, and ocular residual astigmatism were compared. The safety outcomes were then studied intraoperatively and postoperatively. A total of 102 patients with age-related cataract were enrolled. At 3 months postoperatively, UCVA, BCVA, and predictive error were not significantly different between the FLACS and conventional PCS groups. Reduction of refractive astigmatism was higher in FLACS. Postoperative ORA was significant lower in FLACS. Reduction of ORA was higher in FLACS. The intraoperative and postoperative complications were also not significantly different between the two groups. FLACS could effectively change refractive astigmatism and ORA; without more complications than conventional PCS. FLACS’ competitive edge in postoperative ORA may provide better visual quality than conventional PCS in patients with a previous history of CRS.

## Introduction

Cataract is the leading cause of blindness worldwide^1^, and cataract surgery is one of the most commonly performed operations, with more than 30 million operations per year in the United States^2^. Phacoemulsification cataract surgery (PCS) became the standard method of cataract treatment^1^. Advanced cataract techniques such as multifocal intraocular lens (IOL), toric IOL, and femtosecond laser-assisted cataract surgery (FLACS) have been commercially available for over 10 years^3–5^. FLACS enables a well-centered and predetermined capsulotomy compared with the conventional method. This machine-controlled capsulorhexis can minimize IOL tiltation^6,7^, resulting in fewer higher-order aberrations^8^.

Corneal refractive surgery (CRS) is one of the most popular surgeries for correcting refractive errors, especially myopia^9^. In the United States, approximately 800,000 laser in situ keratomileuses (LASIK) procedures were performed annually.

Accurate IOL calculation for cataract surgery after CRS is a major challenge^10^. CRS alters the anterior corneal curvature and anterior to posterior corneal relationships, causing difficulty in predicting the effective lens position^11^. CRS also affects corneal thickness, integrity, and irregularity^12^. Because these changes cause an unpredictable deterioration of visual quality, accurate prediction, and intended outcome are important in patients with a previous history of CRS and must be confirmed.

To date, there has been no clinical research analyzing the refractive and safety outcomes of FLACS after CRS. Only a few case reports suggested the benefit of FLACS in patients with a previous history of CRS^13,14^. This study aimed to evaluate the effect and safety of FLACS after CRS comparing with conventional PCS.

## Results

A total of 102 eyes of 102 patients with age-related cataracts and a previous history of CRS were included in this study. The mean follow-up period was 90.7 days [86–94 days]. Table 1 shows the baseline characteristics of the study population according to FLACS and conventional PCS. The baseline characteristics of FLACS and conventional PCS were not significantly different.

**Table 1:** Baseline Characteristics between FLACS and conventional PCS.

Parameter Mean ± SD [Min, Max]	FLACS (n = 51)	Convention (n = 51)	p value
Age	61.58 ± 9.00 [29, 67]	62.58 ± 10.21 [22, 72]	0.601
Sex (M/F), n	25/26	23/28	0.692
Laterality (R/L), n	29/22	27/24	0.691
Corneal refractive surgery, n (%)			0.376
PRK	10 (19.6)	5 (9.8)	–
LASIK	34 (66.7)	38 (74.5)	–
LASEK	7 (13.7)	8 (15.7)	–
Surgeon, n (%)			0.196
Surgeon 1	9 (17.6)	12 (23.5)	–
Surgeon 2	16 (31.4)	22 (43.2)	–
Surgeon 3	26 (51.0)	17 (33.3)	–
Period between cataract surgery and corneal refractive surgery (years)	16.11 ± 6.80 [2,27]	14.76 ± 5.11 [6, 30]	0.260
UCVA (decimal)	0.26 ± 0.19 [0.1, 0.8]	0.24 ± 0.20 [0.1, 0.8]	0.606
BCVA (decimal)	0.45 ± 0.30 [0.1, 1.0]	0.43 ± 0.25 [0.1, 1.0]	0.715
Preoperative spherical equivalent (D)	− 5.85 ± 4.39 [− 12.25, 4.25]	− 5.53 ± 3.38 [− 10.25, 4.50]	0.681
Preoperative refractive astigmatism (D)	1.12 ± 0.56 [0.25, 3.50]	1.10 ± 0.79 [0.00, 3.50]	0.883
≤ 0.50 D, n (%)	10 (19.6)	9 (17.6)	0.799
≤ 1.00 D, n (%)	25 (49.0)	27 (52.9)	0.692
Preoperative corneal power (D)	39.15 ± 2.07 [36.35, 44.55]	39.29 ± 2.27 [34.00, 42.90]	0.746
Preoperative corneal astigmatism (D)	0.79 ± 0.53 [0.00, 1.2]	0.84 ± 0.49 [0.00, 1.2]	0.622
≤ 0.50 D, n (%)	21 (41.2)	20 (39.2)	0.540
≤ 1.00 D, n (%)	43 (84.3)	39 (72.5)	0.318
Axis group of preoperative corneal astigmatism			0.657
Against-the-rule, n (%)	13	17	–
Oblique, n (%)	10	10	–
With-the rule, n (%)	28	24	–
Preoperative ocular residual astigmatism (D)	1.29 ± 0.77 [0.26, 3.20]	1.20 ± 0.88 [0.21, 3.21	0.584
Axial length (mm)	27.23 ± 2.51 [23.59, 34.86]	27.19 ± 2.34 [23.64, 34.07]	0.934
Anterior chamber depth (mm)	3.60 ± 0.41 [2.96, 4.27]	3.65 ± 0.36 [2.91, 4.36]	0.514

*BCVA* best corrected visual acuity, *D* diopter, *FLACS* femtosecond laser assisted cataract surgery, *PCS* phacoemulsification cataract surgery, *SD* standard deviation, *UCVA* uncorrected visual acuity.

Table 2 shows the 3-month postoperative outcomes with indices of the Alpins method. There were no significant differences between FLACS and conventional PCS in terms of uncorrected visual acuity (UCVA), best-corrected visual acuity (BCVA), and predictive error (p = 1.000, 1.000, and 0.796, respectively). The proportions of preoperative and postoperative refractive astigmatism ≤ 0.50 D were changed from 20 to 49% in FLACS and 18 to 49% in conventional PCS, and the changes in proportion were not significantly different between the two groups (p = 0.787). In the Alpins method, none of the indices differed between the two groups after adjustment for target-induced astigmatism (TIA). Postoperative ORA was 0.20 D (95% CI 0.05–0.35) lower in FLACS than in conventional PCS (p = 0.018).

**Table 2:** Postoperative results at 3 months between FLACS and conventional PCS.

Parameter Mean ± SD [Min, Max]	FLACS (n = 51)	Convention (n = 51)	p value
UCVA (decimal)	0.73 ± 0.28 [0.2, 1.0]	0.73 ± 0.27 [0.2, 1.0]	1.000
BCVA (decimal)	0.96 ± 0.07 [0.8, 1.0]	0.96 ± 0.08 [0.8, 1.0]	1.000
Postoperative spherical equivalent (D)	− 1.12 ± 1.25 [+ 0.75, − 3.25]	− 1.14 ± 1.21 [+ 0.50, − 3.00]	0.935
Prediction error (D)	− 0.30 ± 0.86 [− 1.13, + 0.78]	− 0.25 ± 1.08 [− 1.22, + 0.78]	0.796
Postoperative refractive astigmatism (D)	0.73 ± 0.60 [0, 1.25]	0.76 ± 0.59 [0, 1.50]	0.800
≤ 0.50 D, n (%)	25 (49.0)	25 (49.0)	1.000
≤ 1.00 D, n (%)	42 (82.4)	41 (80.4)	0.799
Postoperative corneal power (D)	39.60 ± 2.01 [36.65, 44.75]	39.23 ± 2.20 [34.55, 42.85]	0.377
Postoperative corneal astigmatism (D)	0.86 ± 0.46 [0.0, 1.2]	0.89 ± 0.51 [0.0, 1.1]	0.756
≤ 0.50 D, n (%)	18 (35.3)	19 (37.3)	0.837
≤ 1.00 D, n (%)	42 (82.4)	43 (84.3)	0.790
Postoperative ocular residual astigmatism (D)	0.63 ± 0.38 [0.05, 1.43]	0.83 ± 0.46 [0.25, 1.65]	0.018†
**Alpins method**			
Target induced astigmatism	0.86 ± 0.46	0.89 ± 0.51	0.756
Surgically induced astigmatism	0.50 ± 0.31	0.53 ± 0.40	0.673
Difference vector	0.89 ± 0.50	0.87 ± 0.51	0.842
Magnitude of error	0.32 ± 0.56	0.29 ± 0.57	0.789
Angle of error	0.2 ± 33.5	− 0.7 ± 32.9	0.891
Absolute angle of error	26.0 ± 18.5	27.6 ± 20.5	0.680
Correction index	0.88 ± 1.01	0.82 ± 0.94	0.757
Index of success	1.28 ± 1.03	1.16 ± 0.98	0.548

*BCVA* best corrected visual acuity, *D* diopter, *FLACS* femtosecond laser assisted cataract surgery, *PCS* phacoemulsification cataract surgery, *SD* standard deviation, *UCVA* uncorrected visual acuity.

^†^*p* < 0.05.

**Figure 1:**
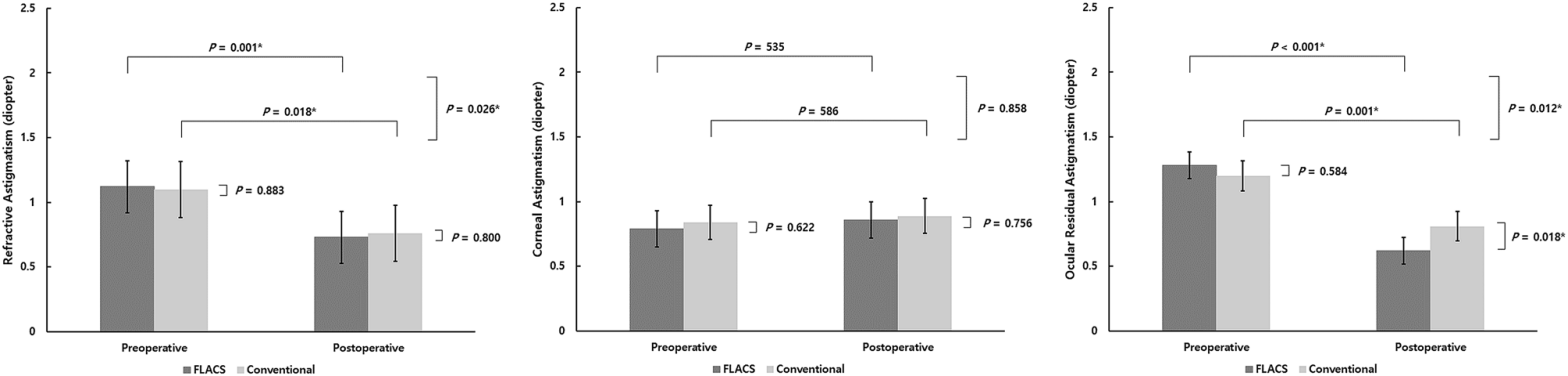
Preoperative and postoperative refractive astigmatism (**a**), corneal astigmatism (**b**), and ocular residual astigmatism (**c**) in FLACS and conventional phacoemulsification cataract surgery. In the process of repeated measure ANOVA, preoperative corneal astigmatism significantly affected refractive astigmatism. *p < 0.05, ^†^adjustment for preoperative corneal astigmatism.

Figure 1 shows the changes in preoperative and postoperative refractive astigmatism and ORA after adjusting preoperative corneal astigmatism. In both FLACS and conventional PCS, postoperative refractive astigmatism was significantly reduced from 1.12 ± 0.56 to 0.86 ± 0.46 (p = 0.001) and 1.10 ± 0.79 to 0.76 ± 0.59 (p = 0.018), respectively. The reduction of refractive astigmatism was significantly higher in FLACS than in conventional PCS (p = 0.026). Postoperative ORA was significantly lower in FLACS than in conventional PCS (p = 0.018). The reduction of ORA was significantly higher in FLACS than in conventional PCS (p = 0.012).

Table 3 shows the intraoperative and postoperative complications according to the groups; no significant differences were observed between the groups (p = 0.727 and 0.647, respectively).

**Table 3:** Intraoperative and postoperative complications of FLACS and conventional PCS.

The complications, n (%)	FLACS (n = 51)	Conventional (n = 51)
≥ 1 intraoperative complications*	5 (9.8)	4 (7.8)
Intraoperative flap complications	0 (0)	0 (0)
Anterior capsule tear	0 (0)	0 (0)
Posterior capsule tear	0 (0)	1 (2.0)
Zonular dialysis	2 (3.9)	2 (3.9)
Intraoperative pupil constriction	2 (3.9)	1 (2.0)
Dropped lens fragments	0 (0)	0 (0)
Suprachoroidal hemorrhage	0 (0)	0 (0)
Incomplete capsulotomy/capsulorrhexis	2 (5.9)	0 (0)
**≥ 1 postoperative complications**^†^	2 (3.9)	3 (5.9)
Postoperative anterior uveitis	0 (0)	0 (0)
Endophthalmitis	0 (0)	0 (0)
Macular edema	1 (2.0)	3 (5.9)
Retinal tear or detachment	1 (2.0)	1 (2.0)
Increased intraocular pressure	0 (0)	1 (2.0)
Vitreous prolapse	0 (0)	0 (0)

*FLACS* femtosecond laser assisted cataract surgery, *PCS* phacoemulsification cataract surgery.

*p = 0.727.

^†^*p* = 0.647.

## Discussion

The results of this study suggest that FLACS for patients with a previous history of CRS was more effective in the change of refractive astigmatism at the 3-month follow-up, which comes from postoperative ORA significantly lower in FLACS than in conventional PCS. FLACS was not accompanied by more complications compared with conventional PCS.

Because the target IOL (D) had been set not only for far vision or emmetropia but also for near vision, UCVA, BCVA, and prediction error were analyzed. UCVA, BCVA, and prediction error were not significantly different between FLACS and conventional PCS. The Barrett True-K formula is one of the most accurate IOL calculation formulas for patients with a previous history of CRS^15^, and the prediction error of the Barrett True-K formula was − 0.24, which was similar to those of our study.

Postoperative ORA was lower, and reduction of refractive astigmatism and ORA were significantly higher in FLACS than in conventional PCS. A multicenter, randomized study showed that FLACS had no advantage in corneal astigmatism, as in our study^16^, but it did not analyze the effect of FLACS on refractive astigmatism and ORA. ORA is composed of posterior corneal astigmatism, lens astigmatism, and retinal astigmatism^17^. The preoperative and postoperative posterior corneal astigmatism was not significantly different in this study. In a previous report, ORA, which also included a part of retinal astigmatism, was found to be inversely correlated with axial length and positively correlated with SE and corneal astigmatism^18^. In our study, axial length, SE, and both anterior and posterior corneal astigmatism were not significantly different between the two groups. Therefore, the difference in ORA between the two groups may be explained by lens astigmatism owing to lens tilt and decentration caused by the different capsulotomy methods between FLACS and conventional PCS^6,7^. Previous studies have reported that horizontal and vertical IOL tilt and decentration were significantly higher in manual capsulotomy and that the results showed a correlation with changes in refraction values between 1 month and 1 year after surgery. IOL tilt and decentration influence visual acuity, dysphotopsia, and coma-like aberrations^19–22^. Moreover, CRS also induced aberrations in a previous study^23^: the root-mean-square wavefront error increased 1.9-fold in a 6.5 mm pupil and significantly in a 3.0 mm pupil, and positive spherical aberration was increased fourfold after myopic LASIK. Oblate corneas that underwent myopic correction benefited from aspheric IOLs with negative spherical aberration, which compensates the positive corneal spherical aberration^24^, and aspheric IOLs are shown to produce more optical quality degradation if tilted or decentered^25^. Considering the visual impacts of the capsulotomy method and CRS, femtosecond laser may provide a higher quality of vision and have a greater impact on patients after CRS.

Trauma vulnerability is a concern in patients with a history of CRS^26,27^. The physical and thermal energy in the process of FLACS could damage the previous CRS-operated tissue^28–30^. However, the complication rates between FLACS and conventional PCS for patients with a history of CRS were not different intraoperatively and postoperatively in our study. The overall intraoperative and postoperative complication rates were 2.8% and 12.5% in FLACS in a previous study^5^, similar to ours. Specifically, posterior capsule tear rates were 0% for FLACS, as in the previous UK reports^5,31^. An intraoperative pupil contracture of 3.9% and incomplete laser capsulotomy of 5.9% were the challenges of FLACS^5,32,33^. There were two incomplete capsulotomy cases in FLACS, and they could be finished with manual capsulorhexis without any complications, their ORAs were not significantly high, within 95% CIs, compared with other FLACS cases. The focus size and pulse separation of laser system are the issue of this complication^34^. The LenSx femtosecond laser platform, which was used in this study, utilizes high energy and low frequency of laser pulse and relatively larger focus size, and this causes larger pulse separation than other femtosecond laser platforms. In this study, 1/102 participants developed postoperative anterior uveitis. In the UK reports, 9.7% of the FLACS group and 8.2% of the PCS group experienced postoperative anterior uveitis, which was higher than our study^5^. The 4-week steroid applied for a month of our protocol may decrease the complication.

There are certain limitations to our study. First, the results of this study did not involve the patients' subjective symptoms. In a Chinese report, FLACS resulted in the dry eye at postoperative day 1, week 1, and month 1^35^. However, in the UK reports, the health-related quality of life and vision questionnaires did not show a significant difference between FLACS and conventional PCS^5,36^. After CRS, there was a possibility of visual disturbance, and other subjective dissatisfaction^37^, and the additional laser treatment could affect the subjective outcomes. Second, our study showed that FLACS had an advantage on ORA and that refractive astigmatism in FLACS was lower than that in conventional PCS after adjustment for preoperative corneal astigmatism. The influence of IOL-induced astigmatism on ORA and aberration was estimated and not directly measured. Further assessment of the patients’ subjective outcomes and analyses for ORA and aberration is required to investigate the effectiveness of FLACS after CRS. Third, the number of detailed complications was too small, and the follow-up period was relatively short to conduct the detailed analysis of FLACS complications. Therefore, we conducted the overall analysis. For example, zonule dialysis was about 4% of patients, which was different from the previous large-scale study, which reported zonule dialysis was about 0.5%^38^. This study could not determine that CRS increases the risk of zonule dialysis because the number of this complication was only one or two.

In conclusion, at the 3-month follow-up, FLACS was effective in terms of the change of refractive astigmatism and ORA. Moreover, FLACS was not accompanied by more complications compared with conventional PCS. The competitive edge of FLACS in postoperative ORA may provide better visual quality than conventional PCS in patients with a previous history of CRS.

## Methods

The Severance Hospital Clinical Research Ethics Committee approved the study protocol (YUHS-SH-IRB-4-2021-0774). The study was conducted in accordance with the tenets of the Declaration of Helsinki, and informed consent was obtained from the subjects after an explanation of the nature and possible consequences of the study.

### Study design and patients

This retrospective comparative study was performed at the Severance Hospital, Yonsei University College of Medicine, between June 2018 and December 2020. The patients with a history of the CRS (LASIK, laser epithelial keratomileusis [LASEK], and photorefractive keratectomy [PRK]) and conducted 3-month follow-up were enrolled. Exclusion criteria were as follows: (a) significant visual impairing diseases such as central corneal opacity, diabetic retinopathy, macular degeneration, and advanced glaucoma, (b) previous history of the complication of CRS (i.e., corneal ectasia), and (c) corneal astigmatism > 1.25 D with any of astigmatism correcting procedures (i.e., arcuate keratotomy and toric IOL insertion). The patients were classified into two groups of 51 patients each: FLACS and conventional PCS. The participants in the conventional PCS group were selected by the computerized matched sampling of age, sex, types of CRS, surgeons, preoperative spherical equivalent and refractive astigmatism, and preoperative corneal power and astigmatism by using Python version 3.8 (https://www.python.org).

### Procedures

All participants underwent a detailed preoperative ophthalmological evaluation, including slit-lamp and fundus examinations. IOL power calculation was performed using optical biometry (IOLMaster 700, Carl Zeiss Meditec AG). Corneal measurement was based on Scheimpflug tomography (Pentacam, Oculus Inc.) simulated K performed within 2 weeks before the surgery. All participants underwent FLACS or conventional PCS with the femtosecond system (LenSx, Alcon Laboratories, Inc.) under topical anesthesia.

The surgical procedures of FLACS and conventional PCS after the femtosecond laser procedure were similar. The clear corneal incision on the temporal side was created using a 2.65–2.80 mm keratome, and the anterior capsule button was removed. In the conventional PCS group, capsulotomy was performed using a capsulotomy needle or forceps. Phacoemulsification was performed using the Centurion vision system (Alcon Laboratories, Inc.). Patients with a single aspherical lens, Tecnis 1-piece intraocular lens (Johnson & Johnson), were enrolled in this study. All operations were performed by experienced surgeons (I.J., K.Y.S., and T.I.K). Postoperative care, including empirical antibiotics and anti-inflammatory medications, was administered as per standard unit practice for cataract surgery for a month.

### Outcomes

The primary outcomes were best-corrected visual acuity (BCVA, decimal scaled), and the refractive outcomes were postoperative spherical equivalent (SE), prediction error, refractive astigmatism, corneal power, corneal astigmatism with vector analysis by the Alpins method in the ACRS website, and ocular residual astigmatism (ORA) at the 3-month follow-up^39^. Prediction error was calculated as the difference of target IOL [diopters (D)] using the Barrett True-K formula and postoperative SE. The vector analysis includes eight indices: TIA, defined as the astigmatic change that the surgery was intended to induce or preoperative astigmatism; surgically induced astigmatism vector (SIA), defined as the astigmatic change that the surgery actually induced; and difference vector (DV), defined as the induced astigmatic change that would enable the initial surgery to achieve its intended target or postoperative astigmatism, the magnitude of error (SIA minus TIA), angle of error (AE, angle between TIA vector and SIA vector), the absolute value of AE, correction index (SIA divided by TIA), and index of success (DV divided by TIA). ORA was calculated using refractive astigmatism and corneal anterior astigmatism values^40,41^. The secondary outcomes were the safety outcomes of intra- and postoperative complications^5^.

### Statistical analysis

The study was framed as a comparative design focusing on BCVA, predictive error, refractive outcomes, and safety outcomes. Because TIA is known to affect SIA and other indices of the Alpins method^42^, a comparative analysis was performed using the Alpins method with the adjustment of TIA. Comparisons of preoperative and postoperative values in each group were analyzed using the paired *t* test. Comparative analyses for change of continuous and categorical values between the two groups were analyzed using the repeated measures ANOVA after adjustment with statistically significant parameters among age, sex, laterality, types of CRS, surgeon, preoperative SE, preoperative corneal power and astigmatism, axis group of preoperative corneal astigmatism, axial length and anterior chamber depth. A p value < 0.05 was considered statistically significant, and a 95% confidence interval (CI) was calculated.
